# Elevated Serum miR-7, miR-9, miR-122, and miR-141 Are Noninvasive Biomarkers of Acute Pancreatitis

**DOI:** 10.1155/2017/7293459

**Published:** 2017-12-03

**Authors:** Pan Lu, Feng Wang, Jia Wu, Cheng Wang, Jing Yan, Zhuo-ling Li, Jia-xi Song, Jun-jun Wang

**Affiliations:** Department of Clinical Laboratory, Jinling Hospital, School of Medicine, Nanjing University, Nanjing, China

## Abstract

**Background:**

It has been reported that several microRNAs (miRNAs), such as miR-141, miR-9, and miR-122, are involved in the regulation of pancreatitis-related proteins or that their levels change in acute pancreatitis (AP) animal models. However, the serum levels, as well as the clinical diagnostic and prognostic values, of these miRNAs in AP patients remain unclear. Furthermore, as a pancreas- (islet) enriched miRNA, miR-7 was reported to be downregulated in AP patients, which requires further verification.

**Methods:**

The levels of miR-7, miR-9, miR-122, and miR-141 were examined and compared using qRT-PCR among 80 severe AP patients, 80 mild AP patients, and 74 healthy controls.

**Results:**

The serum levels of these four miRNAs were increased markedly in the AP patients compared with the controls, and these levels decreased significantly after effective therapy. Particularly, the level of miR-7 was higher in severe AP patients than in mild AP patients. ROC curve analysis demonstrated that four miRNAs could be used as potential biomarkers for AP. Moreover, these miRNAs showed strong positive correlations with CRP, which may be associated with inflammation.

**Conclusions:**

The serum miR-7, miR-9, miR-122, and miR-141 levels were increased in AP patients. These 4 miRNAs may represent diagnostic and prognostic biomarkers for AP.

## 1. Introduction

Acute pancreatitis (AP) is a common pancreatic disease in clinical practice [1, 2]. It has been defined as early activation of pancreatic enzymes in the pancreas, leading to autodigestion of the pancreas [3, 4]. The development of AP is a complex process, which involves many lifestyle and basic disease-related factors such as alcohol, smoking, hypertriglyceridemia, and others [5–7]. Although most AP is mild and self-limited, 10% to 20% of patients experience severe acute pancreatitis (SAP), which is a clinically life-threatening acute abdominal disorder with a mortality rate of approximately 20% [8]. Therefore, early diagnosis and evaluation of AP are critical to enable doctors to prescribe the correct therapies and medicines for patients.

The diagnosis of AP is substantially based on a combination of clinical signs and symptoms, imaging techniques, and laboratory results. Although contrast-enhanced computed tomography is the reference standard for AP diagnosis, it is usually expensive and radioactive [9]. The assessment of pancreatic enzymes, which are released early from necrotic tissue, is the cornerstone of laboratory diagnosis in this clinical setting. Although most current guidelines and recommendations indicate that lipase should be preferred over total and pancreatic amylase, there is no single laboratory or clinical parameter that shows optimal diagnostic accuracy [10–12]. For evaluating the severity of AP, several scoring systems, such as the APACHE II (Acute Physiology and Chronic Health Evaluation II) score and Ranson score, have been used clinically. However, these scoring systems, which include many clinical and biochemical parameters, are complicated and time-consuming. SAP patients usually experience rapid disease progression, poor prognosis, and high mortality. In view of these problems, the application of scoring systems has been limited. Therefore, simple and convenient biomarkers are urgently needed to help diagnose AP at an early stage.

Serum contains large numbers of stable miRNAs derived from various tissues/organs. The serum miRNA expression profile can be used as a novel serum-based biomarker, potentially offering more sensitivity and specificity than the currently available tests for early diagnosis of cancer and other diseases [13]. Accumulating evidence has demonstrated that miRNA expression is mechanistically related to the occurrence and development of AP [14]. This accumulating evidence also provides a new perspective for diagnosis, pathogenesis, and eventual gene therapy in AP. We aimed to identify more sensitive and specific biomarkers for the early diagnosis of AP. Up to now, there have been several studies on miRNAs in animal models and in the clinical aspects of AP. The role of miR-216a in AP has been studied systematically, indicating that miR-216a can be used as a biomarker for the diagnosis of AP. Some miRNAs have been reported to be associated with AP, but their clinical values are still unclear. Han et al. reported that the serum levels of miR-9 increased significantly in a mouse AP model induced by intraperitoneal injection of cerulein [15]. In addition, miR-9 is also expressed at high levels during human pancreatic islet development [16]. However, the serum levels of miR-9 in AP patients and its clinical value have not been reported. The expression of miR-122 was increased in AP rat mesenteric lymph and plasma. Although one study demonstrated that the plasma levels of miR-122 in patients with AP were increased, this study was performed with a very small sample size (only 5 cases in each group), and there was little statistical significance [17]. Moreover, Zhu et al. constructed a mouse AP model to study the role of miR-141 in pancreatitis and explore gene therapy for AP. Their results showed that miR-141 may block the process of autophagosome formation during AP [18]. However, the levels of miR-141 in AP patients remain unknown. Furthermore, miR-7 is the most abundant endocrine miRNA in islets, and its expression is regulated in pancreatic development in humans [16, 19]. Only one clinical study revealed that the serum levels of miR-7 in AP patients are decreased [20], which seems unreasonable and needs to be clarified. Therefore, the changes in miR-7, miR-9, miR-122, and miR-141 in AP patients need to be investigated, and whether these miRNA levels are associated with AP severity and clinical improvement also needs to be clarified.

In the present study, we used the quantitative reverse-transcription polymerase chain reaction (qRT-PCR) assay to assess the levels of serum miR-7, miR-9, miR-122, and miR-141 among MAP, SAP, and healthy control participants. We also evaluated the change in these miRNA levels after AP patient treatment. We also performed receiver operating characteristic curve (ROC) analysis on the selected miRNAs in AP patients and controls enrolled in this study, calculated the area under the ROC curve (AUC), and analysed the correlations between the altered serum miRNAs and haematological indices. We would like to find biomarkers with better sensitivity and specificity to diagnose AP and determine AP severity. We also would like to provide new insight into the physiological traits related to the molecular mechanisms, which has important implications for the prevention and treatment of AP.

## 2. Materials and Methods

### 2.1. Study Design and Subjects

The present study enrolled 160 hospitalized subjects with AP who were treated in the Department of Gastroenterology or Department of General Surgery of Jinling Hospital (Nanjing, China) from January 2016 to January 2017. The exclusion criteria for AP included the presence of malignant tumours, autoimmune disease, diabetes, age of less than 18 years old, and pregnancy. Additionally, 74 subjects who sought routine health check-ups at Jinling Hospital and exhibited no evidence of disease were recruited as the control group.

The diagnosis of AP requires that two of the following three clinical features are met: (1) abdominal pain consistent with AP; (2) amylase and/or lipase levels greater than 3 times the upper limit of normal; and (3) characteristics of AP in an abdominal computed tomography (CT) examination.

In routine practice, multiple prognostic scoring systems, such as the APACHE II score, Ranson score, and Balthazar CT scores, were collected for evaluation of the severity of the disease. SAP is established by either an APACHE II score ≥ 8, Ranson score ≥ 3, Balthazar CT classification for D and E level, the presence of more than one organ failure (more than 48 h), or local complications. Those with an APACHE II score < 8, Ranson score < 3, and Balthazar CT classification for A, B, or C level not accompanied by organ failure and local or systemic complications were classified as having MAP. The treatment of AP varies with disease severity. In clinical treatment, MAP patients only undergo a relatively simple treatment, while SAP patients often need intensive care. Therefore, we selected patients with MAP and SAP to identify AP patients in the early stages of the disease to take appropriate treatment measures in a timely fashion.

All patients and healthy participants provided written informed consent prior to study commencement. The study protocol was approved by the ethics committees of Jinling Hospital. The study was performed in accordance with the 1964 Declaration of Helsinki and the REMARK guidelines for biomarker studies.

### 2.2. Serum Sampling

Serum samples were collected within the first 24 h from the AP patients. For serum sample posttreatment, we collected samples from AP patients whose physical conditions had improved after a period of therapy. In the treatment of AP, conservative internal medicine treatment is the basis for the entire treatment. Conservative treatment included fasting, fluid infusion, oxygen inhalation, gastrointestinal decompression, inhibition of gastric acid and pancreatic secretion, inhibition of trypsin activity, correction of acid-base imbalance and electrolyte disturbances, improvement of microcirculatory disturbances, and nutritional support. The treatment described in the previous sentence is the main treatment of MAP patients. SAP patients, because of their critical condition and the existence of a variety of complications, often need further therapy, such as time in the intensive care unit, interventional radiology, and interventional endoscopy [21]. The serum was separated by centrifugation at 3000 ×g for 10 min and then stored at −80°C until analysis.

### 2.3. RNA Isolation and Reverse Transcription

Total RNA was extracted from 100 *μ*L of serum using 1-step phenol/chloroform purification according to previously described protocols [22]. The resulting RNA pellet was dissolved in 20 *μ*L of DEPC water and stored at −80°C until further analysis. The total RNA (2 *μ*L) was reverse-transcribed into cDNA in a reaction volume of 10 *μ*L using AMV (avian myeloblastosis virus) reverse transcriptase (TaKaRa, Otsu, Japan) and a stem-loop RT primer (Applied Biosystems) under the following conditions: 16°C for 30 min, 42°C for 30 min, and 85°C for 5 min.

### 2.4. Quantitative Real-Time PCR

A hydrolysis probe-based qRT-PCR assay was performed according to the manufacturer's instructions (7300 Sequence Detection System, Applied Biosystems) with a minor modification, as previously described [23]. The PCR conditions were as follows: 95°C for 5 min, 40 cycles of 95°C for 15 s, and 60°C for 1 min. All reactions, including the no-template controls, were conducted in triplicate [24]. Because traditional reference genes, such as U6 and 5S rRNA, are easily degraded in serum samples, they cannot be used as internal controls in our system; thus, to ensure the accuracy of RNA extraction and serve as an external reference, we added MIR2911 (5′-GGCCGGGGGACGGGCUGGGA-3′) to each sample during RNA extraction at a final concentration of 10^6^ fmol/L. Because it has no mammalian homologue, the measurement of MIR2911 expression has high repeatability and reproducibility. The relative miRNA levels were normalized to MIR2911 and were calculated using the comparative Cq method (2^−ΔCq^). ΔCq was calculated by subtracting the Cq values of MIR2911 from the Cq values of the target miRNAs.

### 2.5. Detection of Clinical Parameters in AP Patients

The serum samples from AP patients were collected within 24 hours after AP onset, and the levels of serum amylase (AMY) and lipase (LPS) were determined using the rate method. Ca^2+^, glucose (Glu), triglyceride (TG), and total cholesterol (TC) were determined using the colorimetric method. These six indicators were measured on a VITROS 5600 automatic biochemical analyser. Haematocrit (HCT) levels were measured using XE-2100 analysers (Sysmex, Kobe, Japan), and C reactive protein (CRP) was measured using a MINDRAY BC-5390 with commercial reagents immediately after blood collection. All experimental samples were tested in duplicate.

### 2.6. Statistical Analysis

All statistical analysis was performed using the statistical analysis system software SPSS 18.0 (Chicago, IL, USA). A Kolmogorov-Smirnov test was used to determine the normality of variables. Normally distributed variables were expressed as the mean [standard deviation (SD)]. Variables with skewed distributions were expressed as the median [interquartile range (IQR)] and were log-transformed to normality. miRNA data are presented as the means ± SEM, and the other variables are expressed as the means ± SD. Comparisons of normal variables among three groups were analysed by a one-way ANOVA test. Comparisons of normal variables between two groups were analysed by Student's *t*-test. Comparisons of gender or other categorical variables between groups were analysed by a Chi-square test. Comparisons of skewed variables between groups were analysed by a Mann–Whitney *U* test. Changes in miRNAs from before to after treatment in patients with AP were analysed using paired *t*-test. Receiver operating characteristic (ROC) curves were generated, and areas under the ROC curves (AUCs) were calculated to evaluate the ability of the candidate miRNAs to predict AP. The relationships between the serum miRNAs and clinical parameters were evaluated by Spearman's rank correlation analysis. A *p* value < 0.05 was considered statistically significant.

## 3. Results

### 3.1. The Demographic and Clinical Data of Participants

A total of 160 patients with confirmed diagnosis of AP during the period of January 2016 to January 2017 and 74 control subjects are included in our analysis. The clinical features of the patients and control participants are listed in Table 1. No significant differences were found in age or sex between the AP patients and the control group (*P* > 0.05). Compared with the MAP patients, the levels of CRP and Glu and the APACHEII score and Ranson score were significantly increased in SAP patients (*P* < 0.01), while the levels of Ca^2+^ and HCT were decreased in SAP patients (*P* < 0.001). Compared with the control individuals, the levels of Glu and TG were increased (*P* < 0.001), while HCT was decreased in AP patients (*P* < 0.01).

### 3.2. Expression Levels of the Selected miRNAs in AP Patients

**Figure 1:**
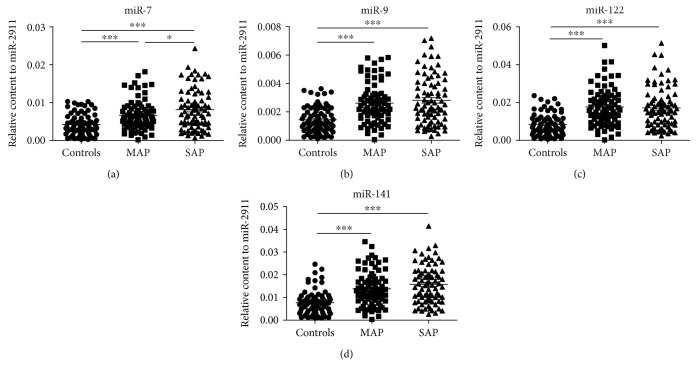
The relative concentrations of miR-7, miR-9, miR-122, and miR-141 in the serum samples from the control (*n* = 74), MAP (*n* = 80), and SAP (*n* = 80) groups (a, b, c, d). Cq values were converted to relative concentrations normalized to MIR2911 values and were calculated using the comparative Cq method (2^−ΔCq^). Each point represents the mean of triplicate samples. ^∗^*P* < 0.05; ^∗∗∗^*P* < 0.001.

We measured the relative serum levels of selected miRNAs by qRT-PCR in all patients and the control individuals (Figure 1). The serum levels of miR-7, miR-9, miR-122, and miR-141 were higher in both MAP and SAP patients than in the controls (all *P* < 0.001). Furthermore, the level of miR-7 was higher in SAP than that in MAP patients (*P* = 0.0436).

### 3.3. ROC Analysis of the Selected Serum miRNAs in AP Patients

**Figure 2:**
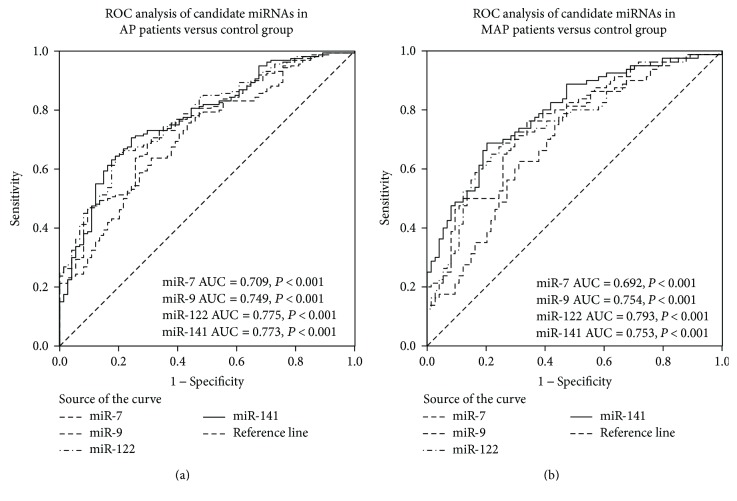
ROC curves to compare the ability of miR-7, miR-9, miR-122, and miR-141 to distinguish AP patients from the healthy controls (a) and MAP from the healthy controls (b).

ROC curve analyses were then conducted to evaluate the diagnostic usefulness of the 4 selected miRNAs for AP (Figure 2). The AUCs of miR-7, miR-9, miR-122, and miR-141 for distinguishing the AP patients from the controls were 0.709 (95%CI, 0.639–0.778; *P* < 0.001), 0.749 (95% CI, 0.685–0.813; *P* < 0.001), 0.775 (95%CI, 0.714–0.836; *P* < 0.001), and 0.773 (95%CI, 0.711–0.835; *P* < 0.001), respectively (Figure 2(a)). The AUCs of miR-7, miR-9, miR-122, and miR-141 for distinguishing the MAP patients from the controls were 0.692 (95%CI, 0.609–0.776; *P* < 0.001), 0.754 (95%CI, 0.678–0.830; *P* < 0.001), 0.793 (95%CI, 0.723–0.863; *P* < 0.001), and 0.753 (95%CI, 0.676–0.829; *P* < 0.001), respectively (Figure 2(b)). It is suggested that these serum miRNAs may be used to distinguish AP patients from the healthy controls.

### 3.4. Alteration of the 4 Serum miRNAs Before and After Treatment

**Figure 3:**
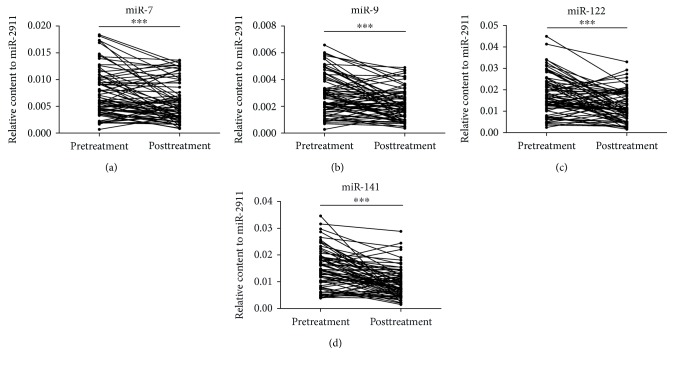
The alteration of the selected miRNAs in serum samples from AP patients before and after treatment ((a, b, c, d) *n* = 71). Cq values were converted to relative concentrations normalized to MIR2911 values and were calculated using the comparative Cq method (2^−ΔCq^). Each point represents the mean of triplicate samples. ^∗∗∗^*P* < 0.001.

Next, to identify the potential of the selected miRNAs to predict clinical improvement, we compared the concentrations of these miRNAs in paired serum samples obtained from 71 patients both before and after treatment. After 2–8 weeks of therapy, the physical condition of these AP patients had improved. The relative content of these 4 serum miRNAs was decreased after treatment (all *P* < 0.001) compared with the values before treatment (Figure 3), which suggested that these 4 serum miRNAs can serve as additional indicators for evaluating AP prognosis.

### 3.5. Correlations between miRNA Concentrations and Clinical Parameters

We then evaluated whether the patients' clinical features were related to miRNA abundance. As shown in Table 2, all four miRNAs had strong positive correlations with CRP (*P* < 0.05). There was no significant relationship between miRNA levels and other clinical parameters (*P* > 0.05).

## 4. Discussion

Current evidence has shown that the levels of some miRNAs are altered in the serum or tissue of rat/mice following acute pancreatitis. Nevertheless, the levels of these miRNAs, such as miR-9 [15], miR-122 [17], and miR-141 [18], in the serum of AP patients are unknown. Moreover, although miR-7 has been studied in AP patients [20], the relationship between this miRNA and the clinical outcome of AP patients has not been reported. Therefore, with the aim of determining the levels of miR-7, miR-9, miR-122, and miR-141 in the serum of AP patients and further evaluating the relationships of these miRNAs with the severity and clinical outcome of these patients, we examined the levels of these 4 miRNAs in the serum of MAP and SAP patients, as well as healthy controls, and analysed their changes in 71 AP patients before and after treatment by qRT-PCR.

Our results revealed that the serum levels of miR-7, miR-9, miR-122, and miR-141 were markedly increased in both SAP and MAP patients. Specifically, miR-7 is associated with AP injury severity. Additionally, these 4 serum miRNAs were significantly increased in AP patients and decreased markedly as the patients attained remission. To our knowledge, this is the first study of the changes in serum miRNAs in paired pretreatment and posttreatment samples from AP patients. Such information will not only increase the number of novel biomarkers for molecular diagnostics and assessment in AP but also provide mechanistic insight regarding the pathogenesis and progression of this disease.

The pathogenesis of AP remains obscure. As it is widely accepted, premature activation of digestive enzymes, which is within the pancreatic acinar cell, leads to autodigestion of pancreatic tissue. This inflammation is followed by infiltration and activation of leukocytes [25]. Recently, several studies have demonstrated an important role for miRNAs in the development and function of AP. Moreover, the expression of some miRNAs in the rat/mouse plasma is altered after AP. For instance, miR-9 has been shown to be significantly upregulated in the serum of AP mice [15]. miR-122 also increased in the AP rodent moderate plasma group compared with the sham group [17]. It has also been reported that miR-7 and miR-9 are highly expressed or specifically expressed in the pancreas [16, 19]. It is known that circulating miRNA results from two processes: active secretion and passive leakage. Thus, we hypothesize that the increased expression of serum miRNAs (miR-7, miR-9, miR-122, and miR-141) in the AP patients may be due to pancreatic cell active or passive release of these miRNAs into the blood. However, further studies are needed to clarify this issue.

In the present study, we also found that the concentrations of these 4 serum miRNAs markedly declined with the clinical improvement of the AP patients. It was suggested that these 4 serum miRNAs may participate in the occurrence, development, and repair of AP. It has been reported that miR-7 exhibits increased expression during development/differentiation of human pancreatic islets, corresponding to the increase observed in insulin transcript levels [16]. The inhibition of miR-7 activates mTOR signalling and promotes adult *β*-cell proliferation in both mouse and human primary islets [26]. Interestingly, Liu and colleagues described that serum miR-7 levels were decreased in AP patients [20], which was inconsistent with our present study. This inconsistency may be due to the different internal parameters in the 2 studies. Liu et al. used hsa-miR-16 as internal control. However, miR-16 was found to be unstable in serum, and it was found to be significantly altered in some diseases [27, 28]. In our study, the serum miRNA levels were normalized to a spike-in exogenous RNA (MiR2911), which is more stable. HMGB1 (high-mobility group box 1) belongs to a family of highly conserved proteins that contain HMG box domains, which play a significant role in inflammation and immunity [29]. As reported, the upregulation of miR-141 could contribute to inhibit HMGB1 expression when AP occurs. Moreover, the level of Beclin-1 is also decreased with the downregulation of HMGB1, which may block the process of autophagosome formation through the HMGB1/Beclin-1 pathway [18]. In our study, we found serum miR-141 upregulation in AP patients, which may be a form of defence response to AP, and miR-141 may be a candidate biomarker of AP. Additionally, it has been reported that the expression of miR-9 in mouse plasma is correlated with the degree of pancreatic fibrosis [15]. miR-122 is positively correlated to insulin biosynthesis [30]. Compared with the sham group, the plasma/mesenteric lymph levels of miR-122 were significantly increased in the AP rat group [17]. However, the role of miR-9 and miR-122 in pancreatitis has not been reported and needs to be further studied. Therefore, we speculate that miR-7, miR-9, miR-122, and miR-141 are critical genomic regulators that may play important roles in AP pathophysiological processes. Further studies are necessary to verify the specific functions of these miRNAs. It was also observed that the levels of the 4 miRNAs were strongly positively related with CRP. CRP is an acute phase protein synthesized by liver cells in response to microbial invasion or tissue damage and is the most sensitive marker of inflammatory disease. This result suggests that the 4 miRNAs may be closely associated with inflammation.

Nevertheless, certain limitations of our study should be mentioned. One of the weaknesses is the relatively small sample size, which may be due to our restrictive selection conditions. Another notable issue is that the study participants were limited to the Chinese Han population, and their serum samples were collected at a single centre, which restricted the generalizability of our findings. Therefore, further large-scale longitudinal cohort studies in multiracial and multiethnic populations are still needed.

## 5. Conclusions

We identified miR-7, miR-9, miR-122, and miR-141 as new candidate biomarkers for AP; these miRNAs show significantly upregulated expression levels in AP patients compared with the healthy controls. Specifically, miR-7 is associated with AP severity. Additionally, the 4 serum miRNAs were decreased markedly as the patients attained remission. These results suggest that these miRNAs have potential as novel markers of AP and may provide an impetus for future evaluation of the clinical value of serum miRNAs in AP pathogenesis and progression. Further research is necessary to verify that these 4 miRNAs are involved in the physiological and pathological processes of AP.

## Figures and Tables

**Table 1:** Demographic and clinical characteristics of the MAP patients, SAP patients, and control group.

Characteristic	MAP (*n* = 80)	SAP (*n* = 80)	Control (*n* = 74)	*p* ^a^	*p* ^b^
Age (y), mean ± SD	42.66 ± 13.46	50.84 ± 14.47	46.18 ± 10.85	0.737	<0.001
F/M, *N*	21/59, 80	29/51, 80	28/46, 74	0.320	0.172
AMY^c^ (U/L)	2.36 ± 0.56	2.38 ± 0.63	—	—	0.807
LPS^c^ (U/L)	2.80 ± 0.64	2.82 ± 0.64	—	—	0.867
CRP (mg/L)	41.20 (10.80–160.00)	143.95 (57.65–239.30)	—	—	<0.001
Ca^2+^ (mmol/L)	2.20 (2.07–2.32)	2.02 (1.87–2.13)	—	—	<0.001
HCT	0.43 (0.38–0.45)	0.36 (0.29–0.42)	0.43 (0.41–0.46)	<0.001	<0.001
Glu (mmol/L)	6.15 (5.00–7.48)	7.55 (5.78–10.23)	4.80 (4.60–5.10)	<0.001	0.002
TG (mmol/L)	1.60 (1.06–3.66)	1.75 (1.10–3.60)	1.06 (0.71–1.38)	<0.001	0.900
TC^c^ (mmol/L)	0.65 ± 0.16	0.63 ± 0.21	0.63 ± 0.06	0.608	0.432
APACHEII score	3.00 (2.00–5.00)	9.00 (8.00–12.75)	—	—	<0.001
Ranson score	1.00 (1.00–2.00)	3.00 (2.25–4.00)	—	—	<0.001

^a^Disease groups versus control group. ^b^MAP versus SAP. ^c^Data were log-transformed before analysis.

**Table 2:** Correlations between miRNA levels in serum and clinical parameters from the AP patient samples.

Variable	miR-7	miR-9	miR-122	miR-141
AMY	*r* = −0.048	*r* = −0.044	*r* = −0.068	*r* = −0.027
	*P* = 0.549	*P* = 0.581	*P* = 0.395	*P* = 0.735

LPS	*r* = −0.012	*r* = −0.007	*r* = −0.034	*r* = 0.025
	*P* = 0.880	*P* = 0.929	*P* = 0.671	*P* = 0.755

Glu	*r* = 0.102	*r* = 0.065	*r* = 0.046	*r* = 0.09
	*P* = 0.201	*P* = 0.420	*P* = 0.566	*P* = 0.262

HCT	*r* = −0.053	*r* = 0.006	*r* = 0.027	*r* = −0.075
	*P* = 0.505	*P* = 0.940	*P* = 0.734	*P* = 0.348

Ca^2+^	*r* = −0.071	*r* = −0.114	*r* = −0.064	*r* = −0.132
	*P* = 0.373	*P* = 0.153	*P* = 0.421	*P* = 0.098

CRP	*r* = 0.215	*r* = 0.189	*r* = 0.235	*r* = 0.222
	*P* = 0.006^∗^	*P* = 0.017*^ζ^*	*P* = 0.003^∗^	*P* = 0.005^∗^

TG	*r* = −0.050	*r* = −0.019	*r* = −0.049	*r* = 0.059
	*P* = 0.535	*P* = 0.816	*P* = 0.540	*P* = 0.463

TC	*r* = 0.007	*r* = 0.004	*r* = 0.009	*r* = 0.003
	*P* = 0.931	*P* = 0.956	*P* = 0.907	*P* = 0.970

^*ζ*^ *P* < 0.05; ^∗^*P* < 0.01.

## Abbreviations

AMV: Avian myeloblastosis virus
AMY: Amylase
AP: Acute pancreatitis
APACHE II score: Acute Physiology and Chronic Health Evaluation II score
AUCs: Areas under the ROC curves
CRP: C-reaction protein
CT: Computed tomography
Glu: Glucose
HCT: Haematocrit
HMGB1: High-mobility group box 1
LPS: Lipase
MAP: Mild acute pancreatitis
miRNAs: MicroRNAs
MSAP: Moderately severe acute pancreatitis
qRT-PCR: Quantitative reverse-transcription polymerase chain reaction
ROC: Receiver operating characteristic
SAP: Severe acute pancreatitis
TC: Total cholesterol
TG: Triglyceride.
